# Evidence-based clinical tool for quantitative analysis of posture in children and adolescents with idiopathic scoliosis

**DOI:** 10.1186/1748-7161-8-S2-O27

**Published:** 2013-09-18

**Authors:** Carole Fortin, Debbie Feldman Ehrmann, Farida Cheriet, Hubert Labelle

**Affiliations:** 1 Centre de recherche, CHU Sainte-Justine, Montreal, Quebec, Canada; 2 École de réadaptation, Faculté de médicine, Université de Montréal, Montreal, Quebec, Canada

## Background

Correction of posture is an important goal of physiotherapy interventions in persons with idiopathic scoliosis (IS) to prevent scoliosis progression and to reduce morphologic deformities and their impact on quality of life. Currently, there are no clinical tools that globally quantify changes in posture attributable to scoliosis progression or treatment effectiveness.

## Purpose

The goal of this study was to develop and validate a new clinical quantitative posture assessment tool among persons with IS.

## Methods

We constructed a software-based program (2-dimensional (2D) tool) to calculate 23 posture indices (PI) representing frontal and sagittal alignment of body segments selected from a literature review[1]. The standing posture of 70 participants aged 10 to 20 years old with IS (Cobb angle: 15º to 60º) was assessed on two occasions by two physiotherapists. Markers placed on several bony landmarks as well as natural reference points (e.g., eyes and ear lobes) were used to measure the PI from photographs with the 2D tool and to calculate 3-dimensional (3D) PI obtained from trunk reconstructions with a surface topography system. Frontal and sagittal Cobb angles and trunk list were also calculated on radiographs. The generalizability theory (f and standard error of measurement – SEM) and Pearson correlation coefficients (r) were used to determine reliability and concurrent validity, respectively.

## Results

In the random design, 21 out of 23 of the PI had a good level of reliability (f ≥ 0.81). The SEM values ranged from 0.9º to 4.3º and 2.1mm to 8.5mm. Correlation between 2D and 3D PI was good to excellent for shoulder, pelvis, trunk list and thoracic scoliosis angles (0.81> r < 0.97; p < 0.01) but fair to moderate for sagittal and thoraco-lumbar/lumbar scoliosis spinal indices (0.30> r <0.56; p < 0.05). Correlation between 2D and radiograph spinal indices was fair to good (-0.33 to-0.80 with Cobb angles and 0.76 for trunk list; p < 0.05).

## Conclusions and discussion

This 2D-imaging tool provides reliable[2] and valid[3] measurements of posture. This new evidenced-based tool may improve physiotherapy practice by facilitating posture analysis. Future longitudinal studies will determine its ability to monitor treatment effectiveness and change in posture over time in persons with IS.
